# How Highly Heterogeneous Sensors with Single‐Molecule Resolution can Result in Robust Continuous Monitoring Over Long Time Spans

**DOI:** 10.1002/advs.202412181

**Published:** 2024-12-24

**Authors:** Chris Vu, Junhong Yan, Arthur M. de Jong, Menno W. J. Prins

**Affiliations:** ^1^ Department of Biomedical Engineering Eindhoven University of Technology Eindhoven 5612 AZ The Netherlands; ^2^ Institute for Complex Molecular Systems (ICMS) Eindhoven University of Technology Eindhoven 5612 AZ The Netherlands; ^3^ Helia Biomonitoring BV Eindhoven 5612 AR The Netherlands; ^4^ Department of Applied Physics and Science Education Eindhoven University of Technology Eindhoven 5612 AZ The Netherlands

**Keywords:** antibody‐based sensing, biosensing, continuous long‐term monitoring, particle‐based sensing, single‐molecule studies

## Abstract

Biomolecular sensors with single‐molecule resolution are composed of multitudes of transducers that measure state changes related to single‐molecular binding and unbinding events. Conventionally, signals are aggregated from many individual transducers in order to achieve sufficient statistics. However, by aggregating signals, transducer‐to‐transducer differences are lost and heterogeneities cannot be studied. Here, transducers with single‐molecule resolution over long time spans are studied, enabling the collection of sufficient statistics from independent transducers. This allows comparisons between transducers that reveal fundamental heterogeneities in their molecular assemblies related to stochastic variations. The study is performed with biosensing by particle motion, a sensing methodology with thousands of particles that dynamically interact with a sensing surface. The signals of individual particles are studied for series of modulations of analyte concentration over 25 h. The results show large differences in individual concentration‐dependent responses. Monte Carlo simulations clarify that heterogeneities can be attributed to stochastic fluctuations in the numbers of binder molecules, and that gradual changes of the response characteristics can be related to losses of molecules in the single‐particle transducers. The results give insights into molecular and temporal heterogeneities of continuous transducers with single‐molecule resolution and explain how sensors can be engineered to achieve robust, precise, and stable biomolecular monitoring.

## Graphical abstract

Methodologies are developed to reveal and understand heterogeneities in continuous biosensors based on a multitude of transducers with single‐molecule resolution. Measurements over long time spans (25 h) show clear inter‐transducer differences, including the shapes of the dose‐response curves. Simulations and time‐dependent changes indicate that the differences are caused by variations in the number of binder molecules per transducer.

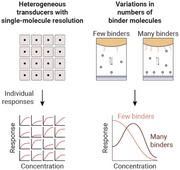

## 1 Introduction

Sensors that continuously monitor concentrations of biomolecules are needed for the development of biochemical‐based monitoring and control strategies in patient care,^[^ ^1^ ^]^ industrial processes,^[^ ^2^ ^]^ and environmental safety and sustainability.^[^ ^3^ ^]^ Metabolite monitoring has been enabled by enzyme‐based methods, such as electrochemical sensors for monitoring glucose in the millimolar concentration range.^[^ ^4^ ^]^ Sensors for continuously monitoring analyte molecules at much lower concentrations (micromolar and below) are being investigated, mainly using reversible affinity‐based binder molecules such as antibodies and aptamers, combined with electrochemical detection,^[^ ^5^ ^]^ fluorescence detection^[^ ^6^, ^7^ ^]^ and particle‐based approaches.^[^ ^8^, ^9^, ^10^, ^11^ ^]^

Biosensors traditionally have macroscopic sensing areas, e.g., an electrode or an optically probed surface area, from which signals are collected that originate from interactions of large ensembles of molecules, without the ability to resolve interactions at the single‐molecule level. In recent years, transducers have been miniaturized and sensors have been developed consisting of arrays of miniaturized transducers, where each individual transducer is small enough to resolve transitions caused by single‐molecular interactions.^[^ ^12^, ^13^, ^14^, ^15^ ^]^ Such sensors enable measurements of low concentrations and also give access to molecular properties that cannot be measured with ensemble‐type sensors.

Here, we describe a methodology to investigate functional heterogeneities in continuous sensors consisting of arrays of transducers with single‐molecule resolution, by exposing the transducers to analyte concentration changes (increasing and decreasing) over long time scales (25 h). The aim is to reveal and understand how the response characteristics differ between transducers as well as how the responses gradually change over long time scales. The methodology is exemplified for biosensing by particle motion (BPM), a continuous biosensing technology based on thousands of biofunctionalized particles that interact with a biofunctionalized sensing surface.^[^ ^10^, ^16^, ^17^ ^]^ The experiments and simulations reveal that the heterogeneities are caused by stochastic fluctuations in the numbers of affinity molecules on particles and surface, and that gradual changes of the response characteristics relate to gradual losses of molecules in the single‐particle transducers. The results show significant molecular and temporal heterogeneities, and give insights in how robust, precise, and stable sensors can be engineered for continuous affinity‐based biomolecular monitoring with single‐molecule resolution.

## 2 Results and Discussion

**Figure 1:**
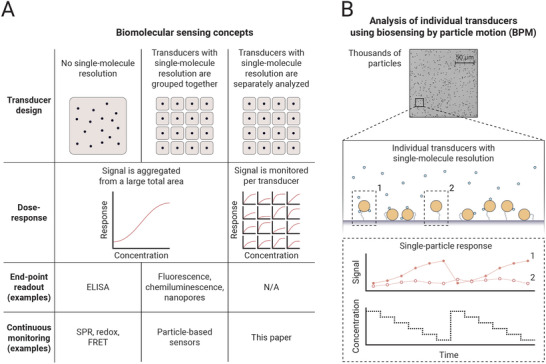
Sensing concepts for translating affinity‐based biomolecular binding into measurement signals. A) Three classes of sensing methods. Left column: ensemble‐based sensing without single‐molecule resolution. Middle column: ensemble‐based sensing with single‐molecule resolution. Right column: transducers with single‐molecule resolution are individually analyzed. B) Transducers with single‐molecule resolution are individually analyzed, exemplified for biosensing by particle motion (BPM). The motions of individual particles are tracked in real‐time using video microscopy. The data is used to detect switching events between bound and unbound states of the particles. The bottom panels exemplify how single‐particle transducers are exposed to concentration‐time profiles over long time spans and how the signals of each particle are measured as a function of time. A sensor is shown with competition format, i.e., the signal increases when the analyte concentration decreases.

**Figure** **1**A describes how affinity‐based biosensors utilize transducers to obtain a sensor signal. A distinction is made between three classes of sensors: i) ensemble‐based transducers, ii) grouping of transducers with single‐molecule resolution, and iii) individual probing of transducers with single‐molecule resolution. Enzyme‐linked immunosorbent assays (ELISA) are a gold standard antibody‐based methodology for quantifying analytes in laboratory‐based settings.^[^ ^14^ ^]^ ELISAs utilize endpoint readout based on ensemble sensing, i.e., many analyte molecules and antibody molecules collectively generate a total measurement signal, for example a luminescence intensity. Ensemble‐based sensing is also the standard in continuous biosensing, e.g., surface plasmon resonance,^[^ ^18^ ^]^ redox sensing^[^ ^5^ ^]^ or Förster resonance energy transfer.^[^ ^7^ ^]^

Transduction methods with single‐molecule resolution are able to distinguish and count individual single‐molecular binding and unbinding events. This gives digital signals and a precision that can theoretically approach the limits of Poisson statistics.^[^ ^19^, ^20^, ^21^ ^]^ Single‐molecule resolution has been demonstrated using several transduction methods, e.g., chemiluminescence signals from particle arrays,^[^ ^20^ ^]^ electrical currents through nanopores,^[^ ^22^ ^]^ motion behavior of particles,^[^ ^10^, ^11^ ^]^ and microscopic imaging of transient binding of fluorescent dyes.^[^ ^8^, ^23^ ^]^ The dose‐response relationships are typically established by aggregating single‐molecule data and by grouping signals from many individual transducers. The grouping increases the statistics of the measurement data, however, the averaging process causes information about heterogeneities to be lost, e.g., differences between molecules, differences between molecular interactions, and differences between transducers.

In this paper we delve into the differences between transducers with single‐molecule resolution, in order to study variabilities and understand how highly heterogeneous sensors can result in robust continuous monitoring over long time spans. BPM is used as the model system, see Figure 1B. BPM is a biosensing method that utilizes biofunctionalized micrometer‐sized particles as transducers with single‐molecule resolution. The motion properties of the particles are affected by reversible affinity‐based single‐molecular interactions between particle and sensing surface. In the experiment, the particles are attached to a substrate via a flexible double‐stranded DNA tether, confining each micrometer‐sized particle to a sub micrometer‐sized area on the sensor surface. The motion behavior of each particle is measured in real‐time using widefield optical microscopy. Further details on the BPM sensing principle can be found in Supporting Information S2.

### 2.1 Probing Transducers with Single‐Molecule Resolution in BPM

**Figure 2:**
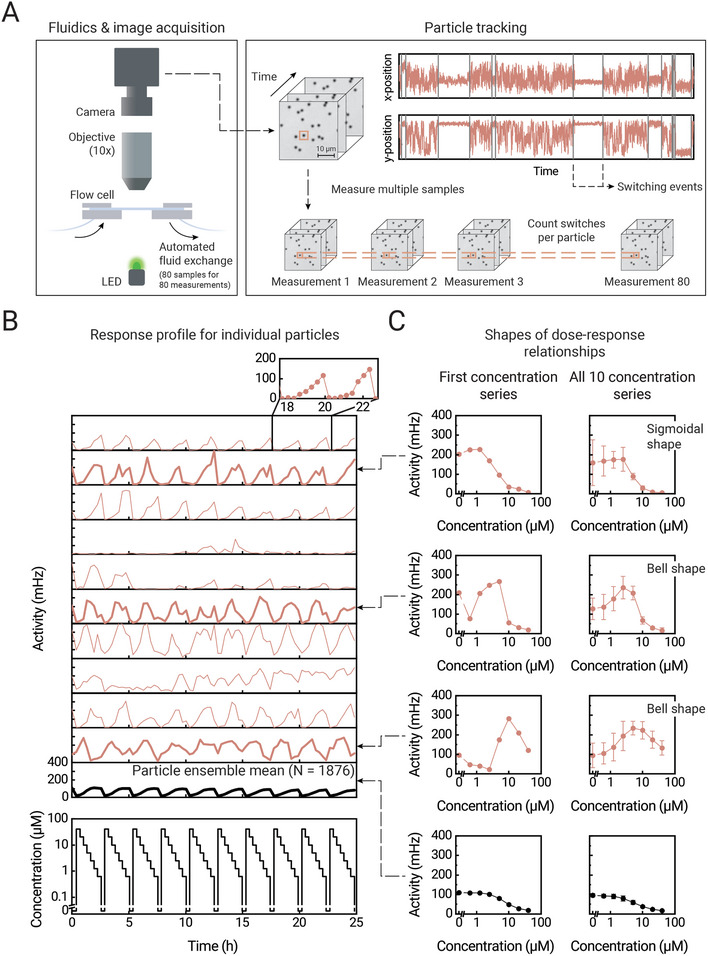
Probing individual particles in a BPM sensor in order to obtain single‐particle responses over a period of 25 h. A) Image acquisition and signal processing in BPM. B) Responses of a selection of individual particles. The bottom panel shows the applied analyte concentration as a function of time. The top panel shows the responses of individual particles to the different analyte concentrations. The particle ensemble mean response is shown as a black curve. In the top panel, a zoom‐in shows how curves are composed of individual data points. C) Examples of dose‐response relationships of individual particles, shown for the first concentration measurement series (between 0 and 2.5 h only) and for the average over all ten concentration series (the total period from 0 to 25 h). The particle ensemble mean response is shown in the bottom graphs. Error bars represent the standard deviations of the data from the ten concentration series.

**Figure** **2**A shows the experimental setup used in this work. The particles are tethered to a surface within a flow cell, that is connected to a custom‐made fluidic system for automated fluid exchange over long time spans. We performed measurements with a competition‐based BPM sensor designed for the continuous monitoring of small molecules (glycoalkaloids), see Supporting Information S2. In short, the sensing surface is functionalized with analyte‐analogue molecules, and the particles are functionalized with antibodies. The antibodies reversibly bind to analyte molecules in solution and to analyte‐analogue molecules on the sensing surface. The binding of a particle to an analyte‐analogue molecule leads to a decrease in the motional freedom of the particle, i.e., the particle switches from an unbound to a bound state. Video images of the particles are processed in real‐time to localize the x‐ and y‐positions of each particle as a function of time. The motion trajectories are then analyzed to determine switching events and the corresponding time‐dependent states of the particles. The fact that every particle is confined to a small area on the surface means that the particles can be identified, recognized, and tracked over long time spans based on positional data. This allows investigations on how the behavior of individual particles changes upon changing the conditions of their environment, e.g., the analyte concentration in solution.

Figure 2B shows an experiment wherein 10 series of analyte concentration‐time profiles were sequentially applied to a single flow cell over 25 h, each series consisting of 8 fluid applications with different analyte concentrations. Particles were tracked during 15 min after each fluid application. The switching activity was measured for each particle individually, which is the frequency of observed switching events. A high switching activity corresponds to many transitions between unbound and bound states of a particle, while a low activity corresponds to few observed transitions. At high analyte concentrations, the activity is low as particles are mainly in the unbound state, since the antibodies are occupied by analyte molecules and rarely bind to analyte‐analogue on the sensing surface. Decreasing the analyte concentration increases the activity, as more antibodies become available to bind to analogue molecules on the surface.

The black curve in Figure 2B shows the particle ensemble mean response, which displays an inverse correlation with the concentration of analyte in solution, as expected for a competition‐based sensor. Interestingly, the responses of individual particles (in red) show a high degree of variation: different particles show behaviors that are clearly different. The differences can be caused by temporal and non‐temporal heterogeneities. Temporal heterogeneities relate to the randomness of timepoints when single particles transition between bound and unbound states. Non‐temporal heterogeneities relate to the physical configurations of the single‐particle transducers, e.g., how the biomolecules are distributed on the particle and surface. We can highlight non‐temporal heterogeneities by plotting the data as time‐averaged dose‐response curves (DRCs) of individual particles. Figure 2C shows the DRCs of three selected particles, for their first concentration series (left) and time‐averaged over all ten concentration series (right). Based on the particle ensemble mean response, one would expect to see sigmoidal‐shaped DRCs. Some particles indeed show sigmoidal‐shaped curves, but surprisingly particles also show bell‐shaped curves. More statistics of measured DRC characteristics can be found in Supporting Information S3. In the next Section, we study which mechanisms could be at the origin of such large particle‐to‐particle differences of DRC characteristics.

### 2.2 Modeling of Particle‐to‐Particle Differences: Time Traces and DRCs

We hypothesize that the observed particle‐to‐particle variations of DRC characteristics can be related to the numbers of binder molecules on particle and substrate. For example, in case of a high number of available binder molecules on particle and substrate, multiple molecular bonds could be formed between particle and surface (i.e., multivalent binding), leading to long particle bound‐state lifetimes and few switching events between bound and unbound states. On the other hand, low numbers of binder molecules could lead to particles that remain mostly in the unbound state and only rarely transition to the bound state.

**Figure 3:**
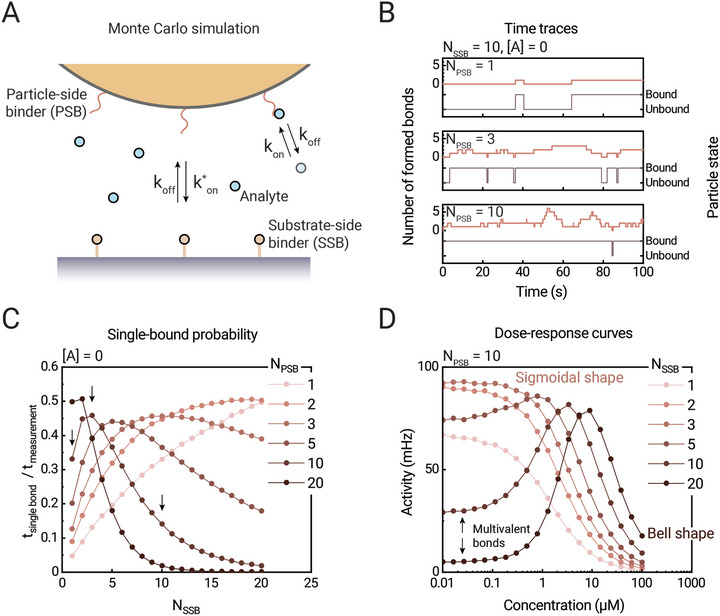
Simulations of BPM particles with different numbers of particle‐side binders (*N*_PSB_) and substrate‐side binders (*N*_SSB_). A) Sketch of an interaction area between a particle and a sensing surface. Particle‐side binders (PSB) can bind to analyte molecules with an association rate constant *k_on_* (M^−1^s^−1^) and to substrate‐side binders (SSB) with an effective association rate constant k^*^_on_ (s^−1^). Both interactions release with a dissociation rate constant *k_off_* (s^−1^). Individual (un)binding events and particle states are tracked. B) Examples of simulated time traces for different numbers of PSB. The formed binder pairs and the particle state are tracked as a function of time. The number of SSB was set to 10, the concentration of analyte was set to zero. Shown are the number of formed bonds between PSB and SSB (left y‐axis) and the particle state (right y‐axis). The simulation input parameters are given in Supporting Information S4. C) Averaged fraction of time when only one molecular bond was present between particle and surface, simulated for varying numbers of PSB and SSB. The arrows correspond to the simulation conditions shown in panel B. D) Simulated dose‐response curves for varying numbers of SSB, for *N*_PSB_ = 10. The data show sigmoidal as well as bell‐shaped DRC characteristics, depending on the number of binder molecules.

**Figure** **3**A sketches a statistical model that we developed to simulate time traces of particle states. A competitive particle‐based transducer is modeled as having *N*_PSB_ particle‐side binders (PSBs) that interact with analyte molecules in solution (concentration [A]) and with *N*_SSB_ substrate‐side binders (SSBs) on the sensing surface. For simplicity, all inter‐molecular binding and unbinding processes are treated as independent Poisson processes, with binding between particle‐side binders and analyte described by rate constants *k_on_* (M^−1^s^−1^) and *k_off_* (s^−1^), and binding between particle‐side binders and substrate‐side binders described by rate constants k^*^_on_ and *k_off_* (both with unit s^−1^). The model is simulated using a numerical Monte Carlo method, as is explained in Supporting Information S4. Figure 3B shows examples of simulated time traces for different numbers of binders on the particle and a constant number of binders on the surface (*N*_SSB_ = 10). Two time‐dependent particle properties are highlighted: the number of formed bonds between PSBs and SSBs (left y‐axis, curve in red) and the observed state of the particle (right y‐axis, curve in gray). The particle state is unbound when there is no PSB‐SSB bond and it is bound when there is at least one PSB‐SSB bond. Figure 3C shows the single‐bound temporal fraction, i.e., the average fraction of time that a particle spends in a single‐bound state when there is only one PSB‐SSB bond. For low numbers of particle‐side binders, the single‐bound temporal fraction increases with increasing numbers of substrate‐side binders. However, for high numbers of particle‐side binders, the fraction decreases because multiple bonds are being formed, as also observed in Figure 3B. The effect on the dose‐response relationship is shown in Figure 3D, with the particle switching activity plotted on the y‐axis. For low numbers of binders, the DRC has a sigmoidal shape, and the curves shift toward the right for higher numbers of binders. At even higher numbers of binders (*N*_SSB_>3 in this example), the shape of the DRCs transitions from a sigmoidal shape to a bell shape. The low switching activities at low analyte concentrations are due to multiple PSB‐SSB bonds being formed between the particle and the substrate, leading to long bound‐state lifetimes of the particles and therefore few state switches per unit time. The simulation results show that the DRC characteristics clearly depend on the available number of binder molecules on particle and substrate, revealing sigmoidal‐shaped and bell‐shaped curves that were also seen in the experimental data of Figure 2.

These results show that observed differences in response characteristics can be attributed to differences in the numbers of binder molecules on particles and surface. Simulations have also been done for Poissonian distributions of binder molecules on particle and substrate, showing that the distributions of DRC characteristics (e.g., the width of the distribution of EC50 values) are similar in simulations and experiments, see Supporting Information S5. Therefore, we attribute the heterogeneities in the particle response characteristics to the stochastics of the molecular assemblies in the particle‐based transducers. In the next Section we proceed to study how the particle response characteristics gradually change over long time scales and how these changes can also be understood from the number of molecular binders in each particle‐based transducer.

### 2.3 How Particle Response Characteristics Gradually Change Over Long Time Scales

The simulations of the previous Section give support for the binder‐heterogeneity hypothesis, i.e., the hypothesis that different particles have different response characteristics (sigmoidal shape vs. bell shape) due to different numbers of binder molecules on particle and substrate. Here, we further investigate the binder‐heterogeneity hypothesis by studying how the particle response characteristics change over long time scales. We use a recently established insight that binder molecules in a competition‐based BPM sensor gradually dissociate from particles and substrate.^[^ ^24^ ^]^ This phenomenon gives an opportunity to investigate the binder‐heterogeneity hypothesis, by first classifying particles according to their DRC characteristics and then studying how particles change their DRC characteristics as a function of time.

**Figure 4:**
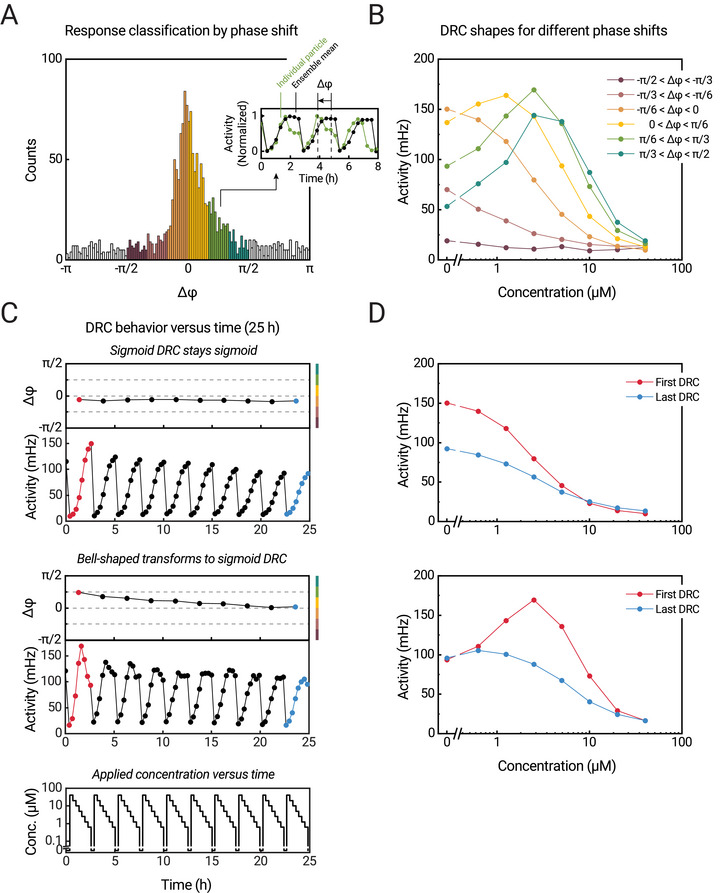
Classification of single‐particle response characteristics, by using the phases of signal‐versus‐time curves. The analysis is applied to the same dataset as in Figure 2. A) To every particle DRC a phase shift Δφ is assigned by Fourier analysis. The phase quantifies the shift of the signal‐versus‐time behavior compared to the ensemble mean, see inset. The DRCs are measured from high to low concentrations, therefore bell‐shaped DRCs give an earlier decrease of signal compared to the ensemble mean, leading to a positive phase shift. Panel A shows a histogram of measured particle phase shifts for the first DRCs (0–2.5 h). The particles are binned in six categories based on their calculated phase shift, as indicated by the six colors ranging from dark red to blue. B) DRCs of averaged particle responses based on different phase‐shift bins from the first applied concentration series. C) Averaged DRC behavior of particles in different phase‐shift bins (top panel: −*π*/6 < Δφ < 0, middle panel: *π*/6 < Δφ < *π*/3). The phase shift plots show the phase shift determined by performing a Fourier analysis on the averaged DRC data. The bars to the right of the panels represent the colors of the particle bins in panels A and B. The bottom panel shows the applied analyte concentration as a function of time. Examples of single‐particle responses in different phase‐shift bins are shown in Supporting Information S7. D) First (red) and last (blue) DRCs from the different phase bins corresponding to the responses shown in panel C.

Figure 2B,C show how DRCs are constructed from measured signal‐versus‐time traces, resulting from applied analyte concentration time series. The data shows that the signal‐versus‐time traces of particles with sigmoidal DRCs have different time delays compared to particles with bell‐shaped DRCs. When the analyte concentration goes from high to low, then particles with sigmoidal DRCs have increasing signals at later timepoints than particles with bell‐shaped DRCs, because bell‐shaped DRCs have their maximum signal at higher analyte concentrations. In other words, when applying decreasing analyte concentrations (as in Figure 2B), particles with bell‐shaped DRCs show an earlier decrease in switching activity compared to particles with sigmoidal DRCs. We can use these differences to objectively classify particle response types by determining the Fourier phase shift of the signal time traces, as explained in **Figure** **4**.

Figure 4A shows the distribution of phase shifts of the particles of the experiment in Figure 2. The phase shifts were determined with respect to the ensemble mean, by a Fourier transform per individual particle for each applied concentration series. Further details can be found in Supporting Information S6. Figure 4B shows the average DRCs of particles within different phase‐shift bins, where the bins are indicated with different colors, in both panels 4A and 4B. The data shows that different phase‐shift bins relate to distinct DRC characteristics, ranging from non‐responsive (dark red) to sigmoidal (orange) to bell‐shaped (green). The results in Figure 2C show that the ensemble mean DRC has a sigmoidal shape. This is in agreement with the distribution in Figure 4A, because the distribution is dominated by particles with sigmoidal‐shaped DRCs. From these results we conclude that the phase shift of the time traces in Figure 2B is a suitable parameter for classifying particles according to their DRC characteristics.

The developed classification now gives the opportunity to investigate how particles change their DRC characteristics over long time spans. Figure 4C,D show how particles change their DRC characteristics over 25 h. The top panels relate to particles that initially (at t = 0) were in the orange bin of panels A and B (−*π*/6 < Δφ < 0, sigmoidal DRC). The bottom panels relate to particles that initially were in the green bin of panels A and B (*π*/6 < Δφ < *π*/3, bell‐shaped DRC). In all panels, the red curves indicate the first DRC (measured between 0 and 2.5 h) and the blue curves indicate the last DRC (measured between 22.5 and 25 h). The results in the top panels show that particles with initially sigmoidal DRCs keep the same DRC shape and the same phase shift over 25 h, while the amplitude of the signal decreases over time. In contrast, the bottom panels show that particles with initially bell‐shaped DRCs change their characteristics toward sigmoidal DRCs (with lower phase shifts). These results can be understood from a gradual loss of binder molecules over time. A particle with a sigmoidal DRC is dominated by single‐molecular bonds, so a loss of binder molecules over time would lead to fewer binding events and a lowered switching activity, still with a sigmoidal DRC characteristic. On the other hand, a particle with a bell‐shaped DRC has a high number of binder molecules and multivalent interactions, so a loss of binders would increase the likelihood of having monovalent bonds, therefore shifting the DRC toward a sigmoidal characteristic. The binder loss hypothesis is further corroborated by analyzing particle bound‐state lifetimes and by simulating the responses of particles with the incorporation of a binder loss rate, see Supporting Information S8.

Measurements and simulations in the regime where the binding between particle and surface is dominated by monovalent bonds, can be used to estimate the binder‐molecule loss rate in the sensor. In the monovalent binding regime, the particles have sigmoidal DRCs, and the measured switching activities are observed to gradually decrease at a rate of about 1.6 ± 0.2% per hour (linear fit through blank measurement data, reported as fitted value ± SE of the fit), see Figure 4C. Simulations show that the magnitude of the switching activity in the monovalent regime is proportional to *N*_PSB_∙*N*_SSB_, i.e., the product of the number of binder molecules on the particle and the number of binder molecules on the surface, see Supporting Information S9. This means that the loss rate of the binder number product *N*_PSB_∙*N*_SSB_ is equal to the observed decrease rate of the switching activity. It is not a priori clear which of the binder molecules would show the highest loss rate in the experiment: the biotinylated antibodies coupled to the streptavidin‐coated particles, or the ssDNA‐conjugated analogue molecules hybridized to the ssDNA‐functionalized PLL‐g‐PEG‐coated surface. Therefore, the observed rate of 1.6% per hour represents an upper limit for the biomolecular loss rate of each of the two binder molecule types.

In conclusion, the experiments of Figure 4 support the hypothesis that the heterogeneities of response characteristics of the particle‐based transducers and their time‐dependencies are caused by an underlying heterogeneity of the numbers of binder molecules on particle and surface, originating from the stochastics of binder molecules in the sensor.

## 3 Conclusion

We have presented a methodology to study heterogeneities in a continuous biosensor consisting of thousands of transducers with single‐molecule resolution. This was exemplified using BPM, where individual particles act as individual transducers that exhibit state changes caused by single‐molecular binding and unbinding events. Heterogeneities were studied by subjecting the transducers to series of analyte concentrations over long time spans, in order to reveal the dose‐response characteristics per particle. The results show a wide range of dose‐response relationships, varying from sigmoid‐shape curves to bell‐shaped curves that shift along the concentration axis. Simulations show that the behaviors are determined by the valency of the particle‐surface bonds (monovalent, multivalent), controlled by the number of binder molecules on particle and surface and by the analyte concentration in solution. This interpretation was confirmed experimentally by measuring distributions of DRC characteristics and by studying changes over long time spans.

Transducers with single‐molecule resolution provide unique opportunities to quantify and exploit functional properties that originate from single biomolecular interactions. Bound‐state and unbound‐state lifetimes and association and dissociation rate constants have been determined using particle‐based sensors,^[^ ^8^, ^11^, ^16^ ^]^ nanopores,^[^ ^22^, ^25^, ^26^, ^27^ ^]^ and transiently binding fluorescent probes.^[^ ^23^, ^28^ ^]^ Distributions of lifetimes and rate constants were used to recognize and suppress non‐specific signals^[^ ^8^, ^28^ ^]^ and to realize multiplexed sensor readouts.^[^ ^29^ ^]^ However, these approaches applied aggregation of data from many individual transducers. The present work offers a new dimension by studying thousands of transducers over long time spans, allowing sufficient statistics to be collected from every individual transducer. This enables detailed investigations into transducer‐to‐transducer differences and their origins, such as the underlying distributions of the numbers of binder molecules. The results show on the one hand stochasticities and heterogeneities of transducers, and on the other hand how the aggregation of data from thousands of transducers enables robust continuous monitoring over long time spans. Regarding the topic of generalizability, performing studies over long time spans is relevant for all sensors with single‐molecule resolution, because inter‐transducer differences become clear when sufficient event statistics are collected from every transducer. Furthermore, methodologies to study heterogeneities of numbers of binder molecules are relevant for all sensors that involve multiple binder molecules per transducer.

In literature, the binding kinetics of biofunctionalized colloidal particles have been modeled using methods such as molecular dynamics,^[^ ^30^ ^]^ Brownian dynamics,^[^ ^31^, ^32^, ^33^ ^]^ self‐consistent thermodynamic models^[^ ^34^ ^]^ and Monte Carlo methods,^[^ ^35^, ^36^ ^]^ with a few addressing the question of how multivalent interactions affect binding state lifetimes.^[^ ^33^, ^35^ ^]^ In this work, we modeled the formation and release of biomolecular bonds between particle and surface by describing the system as a discrete number of binder molecules on a particle interacting with a discrete number of binder molecules on a surface and with biomolecules in solution, using effective rate constants for the inter‐molecular interactions, implemented in a numerical Monte Carlo simulation. Non‐specific interactions were ignored and geometric and steric hindrance effects were neglected. Despite these simplifications, the model is able to relate the main experimental observations (shapes of the single‐particle dose‐response curves, sigmoid shape versus bell shape, distributions of DRCs, and DRC changes over long time scales) to the presence of monovalent and multivalent bonds between particle and surface, to variations in the numbers of binder molecules on particle and surface, and to loss rates of the binder molecules. In further research, we will experimentally study the role of non‐specific interactions and develop descriptions that can be added to the simulation model. Furthermore, a Brownian dynamics simulation will be developed in order to understand how effective rate constants originate from molecular properties, particle properties, and geometrical and steric effects.

The methodology of this paper focuses on how functional characteristics of affinity‐based sensors can be investigated, understood, and exploited on a fundamental single‐molecule level. In follow‐up work, we will investigate the properties for different target molecules (small molecules, proteins, nucleic acids), binder molecules (antibodies, Fab fragments, aptamers), coupling strategies (random orientation, site directed coupling), blocking strategies (bottle brush, zwitterionic), sensor formats (competition, sandwich), and different compositions of biological fluids. It will become important to quantify and understand how different molecular designs lead to distributions of monovalent and multivalent interaction characteristics and to different overall sensor functionalities. We believe that the results of this work will pave the way to engineering strategies and design rules for achieving sensors that enable robust, precise and stable biomolecular monitoring over long time spans for a wide range of applications.

## Conflict of Interest

J.Y. and M.W.J.P. are co‐founders of Helia Biomonitoring.

## Supporting information



Supporting Information

## Data Availability

The data that support the findings of this study are available from the corresponding author upon reasonable request.

## References

[1] E. R. Kim , C. Joe , R. J. Mitchell , M. B. Gu , Trends Biotechnol. 2023, 41, 374. 36567185 10.1016/j.tibtech.2022.12.005

[2] M. S. Firouz , K. Mohi‐Alden , M. Omid , Food Res. Int. 2021, 141, 110113. 33641980 10.1016/j.foodres.2021.110113

[3] F. Ejeian , P. Etedali , H.‐A. Mansouri‐Tehrani , A. Soozanipour , Z.‐X. Low , M. Asadnia , A. Taheri‐Kafrani , A. Razmjou , Biosens. Bioelectron. 2018, 118, 66. 30056302 10.1016/j.bios.2018.07.019

[4] W. Gao , G. A. Brooks , D. C. Klonoff , J. Appl. Physiol. 2018, 124, 548. 28970200 10.1152/japplphysiol.00407.2017

[5] A. M. Downs , K. W. Plaxco , ACS Sens. 2022, 7, 2823. 36205360 10.1021/acssensors.2c01428 PMC9840907

[6] A. A. Hariri , A. P. Cartwright , C. Dory , Y. Gidi , S. Yee , I. A. P. Thompson , K. X. Fu , K. Yang , D. Wu , N. Maganzini , T. Faegin , B. E. Young , B. H. Afshar , M. Eisenstein , M. J. F. Digonnet , J. Vuckovic , H. T. Soh , Adv. Mater. 2024, 36, 2304410. 10.1002/adma.202304410 37975267

[7] I. A. P. Thompson , J. Saunders , L. Zheng , A. A. Hariri , N. Maganzini , A. P. Cartwright , J. Pan , S. Yee , C. Dory , M. Eisenstein , J. Vuckovic , H. T. Soh , Sci. Adv. 2023, 9, eadh4978. 37738337 10.1126/sciadv.adh4978 PMC10516488

[8] V. Lamberti , M. Dolci , P. Zijlstra , ACS Nano 2024, 18, 5805. 38334312 10.1021/acsnano.3c12428 PMC10883122

[9] P. Zhu , V. A. Papadimitriou , J. E. van Dongen , J. Cordeiro , Y. Neeleman , A. Santoso , S. Chen , J. C. T. Eijkel , H. Peng , L. I. Segerink , A. Y. Rwei , Sci. Adv. 2023, 9, eadf5509. 36753543 10.1126/sciadv.adf5509 PMC9908015

[10] A. D. Buskermolen , Y.‐T. Lin , L. van Smeden , R. B. van Haaften , J. Yan , K. Sergelen , A. M. de Jong , M. W. J. Prins , Nat. Commun. 2022, 13, 6052. 36229441 10.1038/s41467-022-33487-3 PMC9561105

[11] E. W. A. Visser , J. Yan , L. J. van IJzendoorn , M. W. J. Prins , Nat. Commun. 2018, 9, 2541. 29959314 10.1038/s41467-018-04802-8 PMC6026194

[12] J. J. Gooding , K. Gaus , Angew. Chem., Int. Ed. 2016, 55, 11354. 10.1002/anie.201600495 27444661

[13] Y. Wu , D. Bennett , R. D. Tilley , J. J. Gooding , Adv. Mater. 2020, 32, 1904339. 10.1002/adma.201904339 31566291

[14] Z. Farka , M. J. Mickert , M. Pastucha , Z. Mikušová , P. Skládal , H. H. Gorris , Angew. Chem., Int. Ed. 2019, 59, 10746. 10.1002/anie.201913924 PMC7318240 31869502

[15] Q. Huang , N. Li , H. Zhang , C. Che , F. Sun , Y. Xiong , T. D. Canady , B. T. Cunningham , Lab Chip 2020, 20, 2816. 32700698 10.1039/d0lc00506a PMC7485136

[16] J. Yan , L. van Smeden , M. Merkx , P. Zijlstra , M. W. J. Prins , ACS Sens. 2020, 5, 1168. 32189498 10.1021/acssensors.0c00220 PMC8177406

[17] C. Vu , Y.‐T. Lin , S. R. R. Haenen , J. Marschall , A. Hummel , S. F. A. Wouters , J. M. H. Raats , A. M. de Jong , J. Yan , M. W. J. Prins , Anal. Chem. 2023, 95, 7950. 37178186 10.1021/acs.analchem.3c00628 PMC10209984

[18] Handbook of Surface Plasmon Resonance, (Ed.: R. B. M. Schasfoort ), The Royal Society of Chemistry, Croydon 2017.

[19] J. Todd , B. Freese , A. Lu , D. Held , J. Morey , R. Livingston , P. Goix , Clin. Chem. 2007, 53, 1990. 17890441 10.1373/clinchem.2007.091181

[20] D. M. Rissin , C. W. Kan , T. D. Campbell , S. C. Howes , D. R. Fournier , L. Song , T. Piech , P. P. Patel , L. Chang , A. J. Rivnak , E. P. Ferrell , J. D. Randall , G. K. Provuncher , D. R. Walt , D. C. Duffy , Nat. Biotechnol. 2010, 28, 595. 20495550 10.1038/nbt.1641 PMC2919230

[21] Y. Zhang , H. Noji , Anal. Chem. 2017, 89, 92. 28105823 10.1021/acs.analchem.6b04290

[22] R. Wei , V. Gatterdam , R. Wieneke , R. Tampé , U. Rant , Nat. Nanotechnol. 2012, 7, 257. 22406921 10.1038/nnano.2012.24

[23] T. Chatterjee , A. Knappik , E. Sandford , M. Tewari , S. W. Choi , W. B. Strong , E. P. Thrush , K. J. Oh , N. Liu , N. G. Walter , A. Johnson‐Buck , Proc. Natl. Acad. Sci. U.S.A. 2010, 28, 595. 10.1073/pnas.2008312117 PMC7502736 32868420

[24] S. Cajigas , A. M. de Jong , J. Yan , M. W. J. Prins , ACS Sens. 2024, 9, 3520. 38967449 10.1021/acssensors.4c00107 PMC11287755

[25] J. P. Fried , Y. Wu , R. D. Tilley , J. J. Gooding , Nano Lett. 2022, 22, 869. 35089719 10.1021/acs.nanolett.1c03976

[26] J. D. Spitzberg , A. Zrehen , X. F. van Kooten , A. Meller , Adv. Mater. 2019, 31, 1900422. 10.1002/adma.201900422 30941823

[27] W. Shi , A. K. Friedman , L. A. Baker , Anal. Chem. 2017, 89, 157. 28105845 10.1021/acs.analchem.6b04260 PMC5316487

[28] A. Johnson‐Buck , X. Su , M. D. Giraldez , M. Zhao , M. Tewari , N. G. Walter , Nat. Biotechnol. 2015, 33, 730. 26098451 10.1038/nbt.3246 PMC4559481

[29] R. M. Lubken , A. M. de Jong , M. W. J. Prins , Nano Lett. 2020, 20, 2296. 32091908 10.1021/acs.nanolett.9b04561 PMC7252944

[30] T. I. N. G. Li , R. Sknepnek , R. J. Macfarlane , C. A. Mirkin , M. O. de la Cruz , Nano Lett. 2012, 12, 2509. 22458569 10.1021/nl300679e

[31] C. B. Korn , U. S. Schwarz , J. Chem. Phys. 2007, 126, 095103. 17362131 10.1063/1.2464080

[32] P. Robert , A. Nicolas , S. Aranda‐Espinoza , P. Bongrand , L. Limozin , Biophys. J. 2011, 100, 2642. 21641309 10.1016/j.bpj.2011.04.011 PMC3117183

[33] R. Lanfranco , P. K. Jana , L. Tunesi , P. Cicuta , B. M. Mognetti , L. di Michele , G. Bruylants , Langmuir 2019, 35, 2002. 30636419 10.1021/acs.langmuir.8b02707

[34] P. Varilly , S. Angioletti‐Uberti , B. M. Mognetti , D. Frenkel , J. Chem. Phys. 2012, 137, 094108. 22957556 10.1063/1.4748100

[35] W. B. Rogers , T. Sinno , J. C. Crocker , Soft Matter 2013, 9, 6412.

[36] M. R. W. Scheepers , S. R. R. Haenen , J. M. Coers , L. J. van IJzendoorn , M. W. J. Prins , Nanoscale 2020, 12, 14605. 32614022 10.1039/d0nr03125a

