# Supplementary material for: How Highly Heterogeneous Sensors with Single‐Molecule Resolution can Result in Robust Continuous Monitoring Over Long Time Spans
Source: Adv Sci (Weinh). 2024 Dec 24;12(7):2412181. doi: 10.1002/advs.202412181 (PMC11831471; doi:10.1002/advs.202412181)
Supplement: Supplementary file 1 — Supporting Information [file ADVS-12-2412181-s001.pdf]

ADVANCED SCIENCE Open Access

Supporting Information

for *Adv. Sci.*, DOI 10.1002/advs.202412181

How Highly Heterogeneous Sensors with Single-Molecule Resolution can Result in Robust Continuous Monitoring Over Long Time Spans

*Chris Vu, Junhong Yan, Arthur M. de Jong and Menno W. J. Prins**

How Highly Heterogeneous Sensors with Single-Molecule Resolution Can Result in Robust Continuous Monitoring over Long Time Spans

Chris Vu[1,2], Junhong Yan[3], Arthur M. de Jong[2,4] and Menno W. J. Prins[1,2,3,4]

[1]Department of Biomedical Engineering, Eindhoven University of Technology, the Netherlands.
[2]Institute for Complex Molecular Systems (ICMS), Eindhoven University of Technology, the Netherlands.
[3]Helia Biomonitoring, Eindhoven University of Technology, the Netherlands.
[4]Department of Applied Physics and Science Education, Eindhoven University of Technology, the Netherlands.

Supporting Information

S1. Experimental Details 2
BPM measurements 2
Image recording and data analysis 2
S2. Biosensing by Particle Motion (BPM) for glycoalkaloid detection 3
S3. Response profiles of individual particles 5
S4. Monte Carlo model of BPM state switching 6
Description of the model 6
Simulation steps 7
Selection of kinetic parameters by analyzing state lifetimes 8
S5. Explaining variations in experimental particle responses 10
S6. Classification of individual particle responses by Fourier analysis 12
S7. Examples of individual particle responses in different phase-shift bins 13
S8. Changes of binding valency over long time spans 14
S9. Dependency of activity on the number of binder molecules 16
S10. References 17

S1. Experimental Details

BPM measurements

Flow cells and particles were prepared as described in previous work.[1] In short, fluidic slides (60 μL in volume, μ Slide III 3in1, ibidi GmbH) were cleaned, sealed and functionalized with an anti-fouling polymer mixture (PLL-g-PEG/PLL-g-PEG-N_3). The slides were subsequently functionalized with dsDNA tether molecules (modified with DBCO on one end and biotin on the other) and ssDNA capture molecules (modified with DBCO) that allowed for the coupling of the analyte-analogue molecule. Streptavidin-coated particles (Dynabeads MyOne Streptavidin C1, Thermo Scientific) were functionalized with 250 nM of anti-solanidine antibodies in a 1:1 (antibody:particles) volume ratio and blocked with 10 μM of polyT (5' biotin—TTT TTT TTT TTT TTT T—3'). Samples containing analyte were prepared by diluting α-solanine (1652 S, Extrasynthese, France) in phosphate buffered saline (PBS) supplemented with 500 mM NaCl (pH 7.4) to achieve concentrations of 40, 20, 10, 5, 2.5, 1.25 and 0.625 μM.

Sample injection into the flow chambers was done using a custom-made fluidic setup, comprised of an automated syringe pump connected to a drip chamber for individual sample injections and a rotary valve for series of sample injections. The system was controlled using a custom-made MATLAB application. Functionalized particles were diluted to a concentration of 90 μg/mL, injected into the flow chamber via the drip chamber and allowed to interact with the dsDNA tethers, after which the particles were further blocked with 100 μM of mPEG-biotin (1 kDa). The system was activated by injecting 5 nM of analyte-analogue (solanidine-ssDNA conjugates) into the flow cell, which could hybridize with the ssDNA capture molecules on the substrate. Residual analyte-analogue was removed via flushing with buffer (PBS supplemented with 500 mM NaCl, pH 7.4), after which samples containing analyte were injected via the rotary valve. For every sample, 200 μL was injected into the flow chamber at a flow rate of 100 μL/min. No flushing was performed in between analyte samples.

Image recording and data analysis

Tracking of tethered particles in flow chambers was done on a custom-made optical setup at a total magnification of 10× using a Grasshopper camera (Point Grey Research Grasshopper3 GS3-U3-23S6M, 1920 × 1200, pixel format: 8 raw, gain 10) in bright field illumination conditions. The positions of the particles were recorded for 15 minutes in a field of view of 707 × 530 μm^2 at a frame rate of 30 Hz with an exposure time of 1 ms. The particles were localized using phasor-based localization, after which the xy-trajectories were used to detect particle switching events using a change-point detection algorithm, which has been described by Bergkamp et al..[2] Tracking of response data per individual particle was done using post-processing with a custom MATLAB script.

S2. Biosensing by Particle Motion (BPM) for glycoalkaloid detection

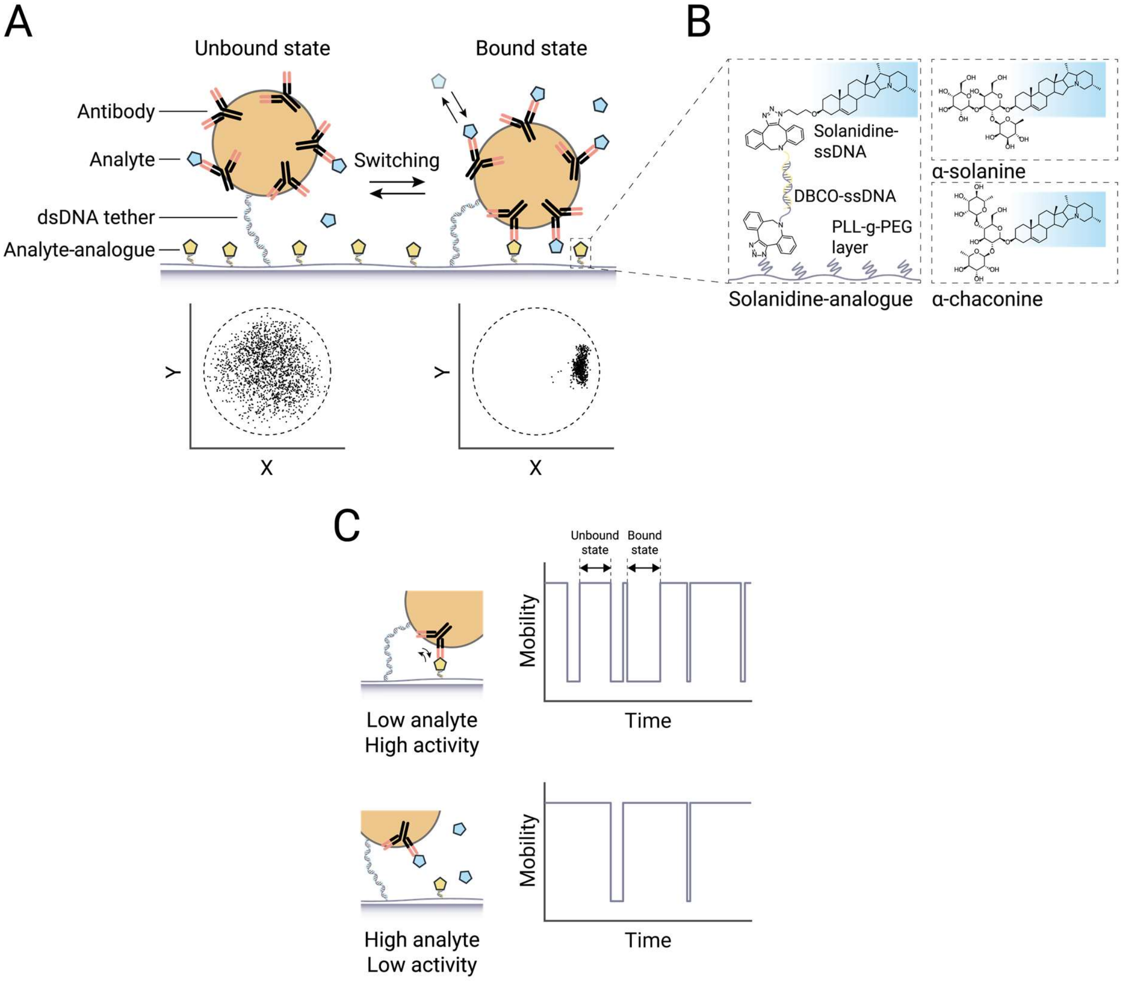

Supporting Figure S1 | Biosensing by Particle Motion (BPM) for glycoalkaloid detection. (A) Particles switch between unbound and bound states. Top: Micrometer-sized particles (orange) are tethered to the sensor surface via double-stranded DNA. Particles are functionalized with antibodies that are specific to the analyte molecules in solution and to analyte-analogue molecules that are attached to the sensing surface. The transient binding of particle-side antibodies to the substrate-side analyte-analogue molecules limits the Brownian motion of the particle. Bottom: Motion patterns are time-aggregated projections of the center of the particle in the xy-plane. The motion patterns differ strongly for the bound and unbound states. (B) Analyte-analogue design for the BPM sensor for glycoalkaloid detection. The analogue consists of solanidine conjugated to single stranded DNA (ssDNA). The oligonucleotide can hybridize with ssDNA molecules that are provided on the sensing surface. Particles that are functionalized with anti-solanidine antibodies can bind to the solanidine moiety of the analogue, but also with the solanidine moieties of glycoalkaloids in solution. In this paper, α-solanine was measured. (C) Sketch of the signals of the BPM sensor. Digital switching events between unbound and bound states are identified by tracking the mobility of the particle over time. Low and high concentrations of analyte in solution result in a high and low frequency of switching events, respectively.

Biosensing by Particle Motion (BPM) is a biosensing technology that relies on detecting state transitions of micrometer-sized particles (Figure S1). In a competition-based BPM sensor (Figure S1A), the particles are provided with binder molecules (in this case antibodies) and the substrate is provided with analyte-analogue molecules. The binder molecules transiently bind to the analogue molecules and to analyte molecules in solution. Particles that do not interact with analogue molecules exhibit a large motion pattern, that relates to the flexibility of the

double-stranded DNA tether. The motion of the particles becomes more confined upon binding of the antibodies to the analogue molecules. The changes in motion are recorded using widefield video microscopy. Figure S1B sketches the molecular design of the analyte-analogue in the competition BPM sensor for glycoalkaloid detection. Here, the functional group of the analyte-analogue molecule is the solanidine moiety. The BPM glycoalkaloid sensor uses anti-solanidine antibodies that recognize the analyte-analogue and the α-solanine in solution. Figure S1C illustrates the mobility of a particle over time at low (top) and high (bottom) concentrations of analyte. A high frequency of binding and unbinding events, i.e., a high switching activity, is observed when no analyte is present. The activity decreases with higher analyte concentrations, as the analyte blocks the binding sites of the antibodies and in that way decreases the probability that the particle can bind to the substrate.

S3. Response profiles of individual particles

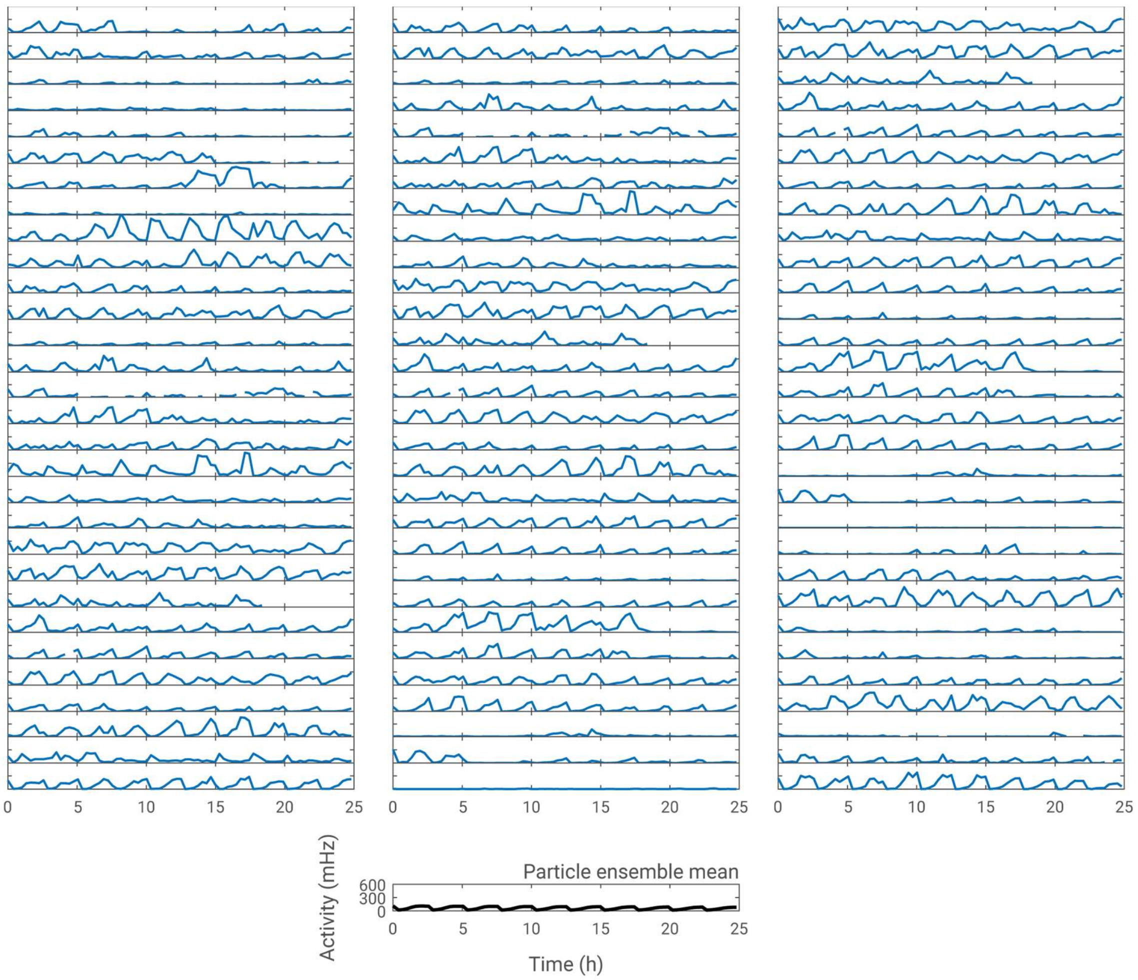

Supporting Figure S2 | Response profiles of individual particles. Each blue curve corresponds to the response of an individual particle. The shown particles (90 in total) were randomly selected from the same dataset as in Figure 2. The black curve in the bottom graph represents the particle ensemble mean (N = 1876). The axes are the same for all graphs: the x-axes show time (0-25 h) and the y-axes show switching activity (0-600 mHz).

S4. Monte Carlo model of BPM state switching

Simulated time traces of particle states were generated using a Monte Carlo model. In this section, we describe the developed model, the simulation steps, and the kinetic parameters that were determined by analyzing experimental state lifetime data.

Description of the model

The Monte Carlo model is based on a number of particle-side binders (PSB, denoted as N_{PSB}) that can freely interact with substrate-side binders (SSB, denoted as N_{SSB}). We assume that the binding interactions of individual PSBs and SSBs behave according to a Poisson point process with exponentially distributed lifetimes. As such, the individual unbound and bound-state lifetimes per PSB-SSB pair were sampled from an exponential distribution with mean state lifetimes:

$$\tau_{unbound} = \left(k_{on}^{*} \cdot \frac{k_{off}}{k_{off} + k_{on} \cdot [A]}\right)^{-1}, \quad (1)$$

and

$$\tau_{bound} = \left(k_{off}\right)^{-1}, \quad (2)$$

where k^{*}_{on} is the effective association rate constant of PSBs to SSBs in the absence of analyte, k_{off} is the molecular dissociation rate constant, k_{on} is the association rate constant of PSBs to analyte molecules in solution, and [A] is the concentration of analyte. The mean unbound-state lifetime in Equation (1) scales with the fractional occupancy of PSBs by analyte molecules according to the Langmuir isotherm ($\frac{k_{off}}{k_{off}+k_{on}\cdot[A]}$). The particle switching activity and bound fraction were determined based on the observed bound states of the particle. A particle is considered to be in a bound state when at least one PSB-SSB bond is formed. This means that the formation of multiple additional PSB-SSB bonds are not counted as switching events.

The nature of the bonds between particle and surface depends on the numbers of binder molecules (N_{PSB} and N_{SSB}) and on the kinetic parameters. Figure S3 illustrates the temporal fraction of single bonds for different effective association rates k^{*}_{on}. A high k^{*}_{on} (see Figure S3A) results in multivalent bonds between particle and surface, except if only one binder molecule is present on the particle or on the surface. In the case where particle-surface bonds are predominantly multivalent, the probability of having a monovalent bond *decreases* with increasing numbers of binder molecules. A low k^{*}_{on} (see Figure S3C) results in long unbound-state lifetimes, a low probability that a particle is monovalently bound to the surface, and an even lower probability that a particle is a multivalently bound state. In the monovalent regime, the probability of having a monovalent bond *increases* with increasing numbers of binder molecules.

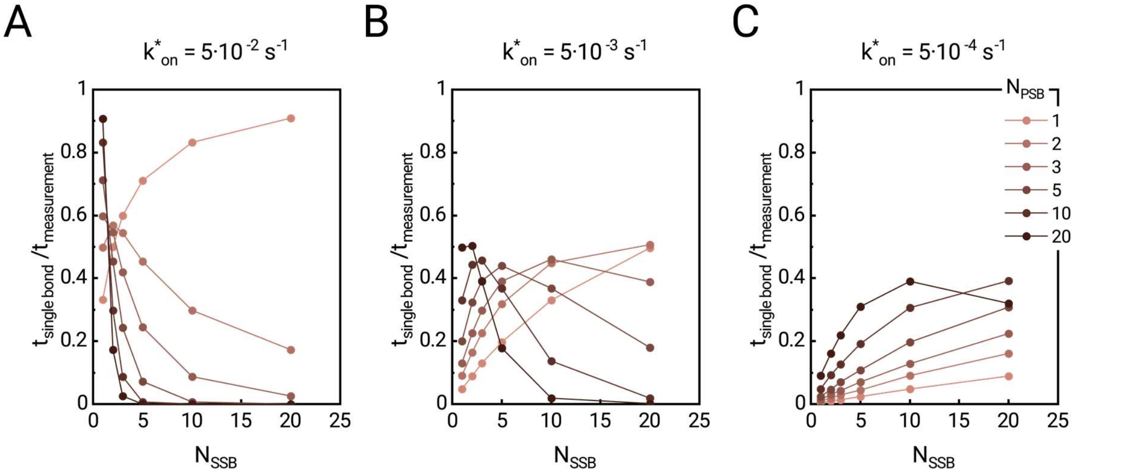

Supporting Figure S3 | Average fraction of time where only one molecular bond was present between particle and surface, simulated for varying numbers of PSB, SSB and effective association rates k^*_{on}: $5{\cdot}10^{-2}$ s^{-1} (A), $5{\cdot}10^{-3}$ s^{-1} (B) and $5{\cdot}10^{-4}$ s^{-1} (C).

Simulation steps

A simulation involves the following steps:

1) A number of parameters are defined prior to starting the simulation, which are given in Table S1.

Table S1 | Input parameters for the Monte Carlo model.

Parameter	Description
k^*_{on}	Effective association rate constant
k_{off}	Molecular dissociation rate constant
k_{on}	Molecular association rate constant
N_{PSB}	Number of particle-side binders
N_{SSB}	Number of substrate-side binders
A	Array consisting of several analyte concentrations
$t_{\mathrm{measurement}}$	Total time within the simulation run
$N_{\mathrm{particles}}$	Number of runs

2) The model first generates a particle with a fixed number of PSB and SSB according to N_{PSB} and N_{SSB}. In simulations where the number of PSB and SSB are generated according to a Poisson distribution (for example as done in Supporting Information S6), N_{PSB} and N_{SSB} instead represent the rate parameters of the Poisson distributions.

3) Per analyte concentration, a mean unbound-state lifetime τ_{unbound} and a mean bound-state lifetime τ_{bound} is determined according to Eq. 1 and Eq. 2, respectively.

4) A time matrix with size $N_{\mathrm{PSB}} \times N_{\mathrm{SSB}}$ is generated containing the unbound-state lifetimes of each SSB with each PSB individually. These lifetimes are generated from a single-exponential distribution with a mean lifetime τ_{unbound}. An additional empty row is added, which is dedicated to the bound-state lifetime of a SSB with a PSB.

5) The simulation is run repeatedly per particle and concentration until the total in-simulation time $t_{measurement}$ has passed for that condition. The in-simulation time is tracked *via* parameter t_{run}. Per iteration, the shortest lifetime within the time matrix is found and subtracted from the other lifetimes.

    a. If this lifetime represented an unbound-state lifetime between a PSB and SSB, all unbound-state lifetimes related to this specific PSB and SSB are removed from the matrix. A bound-state lifetime is generated according to a single-exponential distribution with a mean lifetime τ_{bound} and is stored within the time matrix.
    b. If this lifetime belonged to a bound-state lifetime between a formed PSB and SSB bond, new unbound-state lifetimes for both binders with unbound PSBs and SSBs are generated and are stored within the time matrix.

6) The subtracted lifetime is added to t_{run}, while the number of present PSB-SSB bonds are counted and recorded per t_{run}. If t_{run} exceeds $t_{measurement}$, the simulation run ends and a new run is started with the same N_{PSB} and N_{SSB}, and the next concentration in array *A*. This procedure is repeated $N_{particles}$ times. All conditions throughout this manuscript are simulated 1000 times for 15 minutes.

Selection of kinetic parameters by analyzing state lifetimes

The values for the kinetic constants to produce the data in Figure S3 are shown in Table S2. We selected these values after analyzing the state lifetimes of the BPM system, see Figure S4. Figure S4A and S4B show the cumulative distribution functions (CDFs) of the bound-state and unbound-state lifetimes, respectively, extracted from the first applied concentration series for the same dataset as in Figure 2. The state lifetimes reflect the effective association (unbound-state lifetimes) and dissociation rates (bound-state lifetimes) of particles and substrate. These were obtained after the classification of each binding state using a custom-made MATLAB script.

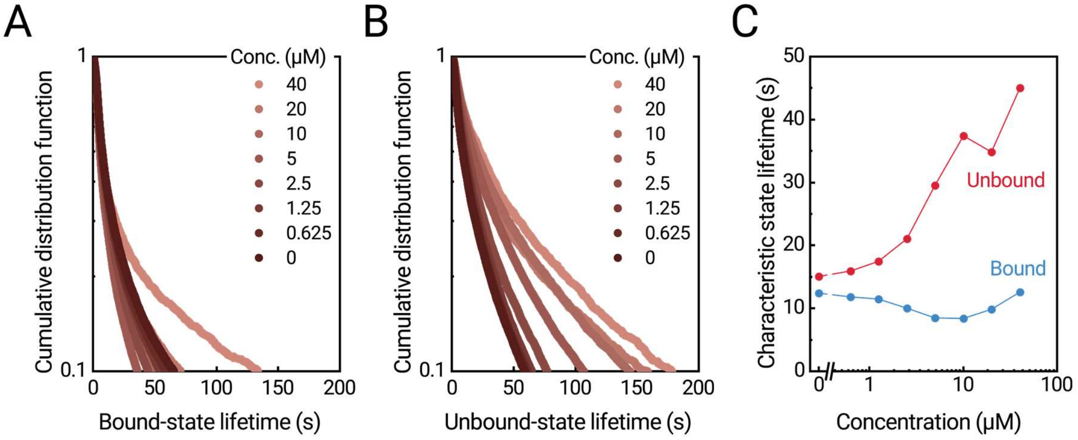

Supporting Figure S4 | Selection of kinetic parameters by analyzing state lifetimes. (A) Bound-state lifetime CDFs extracted from the first applied concentration series of the same dataset shown in Figure 2. (B) Unbound-state lifetime CDFs extracted from the first applied concentration series of the same dataset shown in Figure 2. (C) Characteristic state lifetimes determined from the CDFs of bound-state (blue) and unbound-state (red) lifetimes shown in panels A and B. The characteristic lifetimes were obtained by fitting each CDF with a single-exponential function and extracting the inverse of the fitted rate parameter.

The majority of the data for all bound-state lifetime distributions (initial 70-80% of the data) show a distribution that appears to be independent of the analyte concentration (see Figure S3A). This indicates that the bound states are dominated by monovalent binding interactions. The deviations of the 40 µM curve are attributed to the low number of bound states (N_{bound} = 2589 compared to N_{bound} = 4000 – 25000 for other concentrations), possibly leading to non-specific and/or false positive bound states to appear more prominently in the CDF. Fitting the CDFs of the bound-state lifetimes with a single-exponential curve yields a characteristic bound-state lifetime of about 10 s for all concentrations, see Figure S4C (blue curve). From this, we determined that the dissociation rate constant k_{off} should be on the order of magnitude of 10^{-1} s^{-1}.

The CDFs of unbound-state lifetimes in Figure S3B show a concentration-dependent trend, where the distribution tends to longer lifetimes with increasing analyte concentrations. This is attributed to analyte molecules interacting with antibodies on the particles, which reduces the likelihood of particles interacting the surface. The lifetime analysis yields distributions that appear multiexponential, which can be attributed to heterogeneities in the association kinetics of particles.[1,3] For simplicity, we fitted the CDFs with a single-exponential curve to obtain a characteristic unbound-state lifetime, see Figure S4C (red curve). The effective particle association rate κ relates to the characteristic unbound-state lifetime in the absence of analyte, which is about 15 s. This yields a rate κ of around 0.07 s^{-1}. However, κ is comprised of many effective association rates of individual binders k^*_{on}. Therefore, κ represents an upper limit for k^*_{on} and k^*_{on} is likely much smaller. We tested lower values of k^*_{on} in the simulation model and determined that a value in the order of 10^{-3} – 10^{-2} s^{-1} gives good correspondence to the experimental data.

Table S2 | Parameter values used in the Monte Carlo simulations in Figure 3.

Parameter	Value	Description
k^*_{on}	$5{\cdot}10^{-3}$ s^{-1}	Effective association rate constant
k_{off}	0.1 s^{-1}	Molecular dissociation rate constant
k_{on}	10^5 M^{-1} s^{-1}	Molecular association rate constant

S5. Explaining variations in experimental particle responses

We quantified the variability in particle responses by analyzing the concentration of halfway responses of individual particles, i.e., the effective concentration of 50% response, called the EC50. The EC50 was determined for dose-response curves with analyte concentration on the x-axis and bound fraction on the y-axis. The bound fraction of a particle relates to the fraction of time a particle spends in the bound state over the total measurement time.[4] In this comparative study, the activity signal is not used as a readout parameter, because the activity dose-response curves give inconclusive EC50 values in case of bell-shaped curves. Bound-fraction DRCs are always sigmoidal (see below) and give well-defined EC50 values.

Figure S5A shows experimental bound-fraction DRCs. A high bound fraction is seen for low concentrations of analyte, as particles interact strongly with the surface. A low bound fraction is seen for high analyte concentrations since particles cannot bind to the surface because the antibodies are occupied by analyte from solution. Bound-fraction EC50s were obtained by fitting the bound-fraction DRCs of individual particles with 4-parameter logistic fit $y = a + (b - a) \cdot x^n/(x^n + EC50^n)$. This equation, also known as the Hill equation, is used to describe receptor-ligand interactions.

Figure S5B shows the distribution of EC50 values of the particles of the experiment in Figure 2 (shown in red). We investigated the origin of the variations by comparing the experimental EC50 distribution to simulation results with a distribution in the numbers of binder molecules. Distributions of binder molecules on a surface can be random, clustered, or dispersed.[5] For simplicity, distributions on particle and surface are assumed with complete spatial randomness (CSR). The CSR hypothesis states that the placement of points within a region follows a Poisson distribution if the process is completely random. Numbers of binder molecules were assigned to particles and surface according to Poisson distributions with rate parameters λ_{PSB} and λ_{SSB}. The results in Figure S5B show that the width of the EC50 distribution of the simulated data (blue) is very similar to the width obtained experimentally (red). This indicates that the heterogeneities in the single-particle response characteristics can be attributed to stochastic variations in the number of binder molecules on the particles and surface.

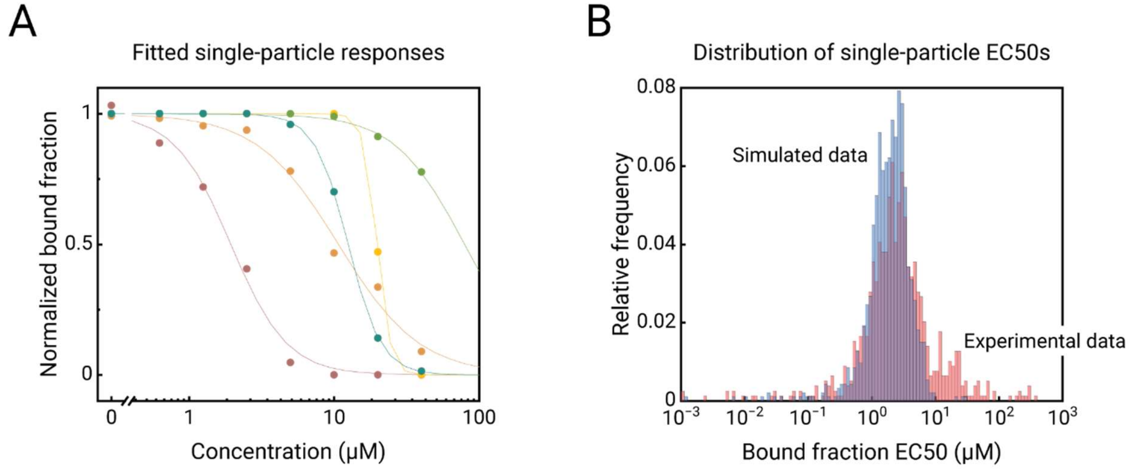

Figure S5 | Explaining variations in experimental particle responses. (A) Examples of measured single-particle bound fraction DRCs. Lines indicate 4-parameter logistic fits to extract the EC50: $(y = a + (b - a) \cdot x^n / (x^n + EC50^n))$. (B) Distributions of single-particle EC50s obtained from the first DRC of the dataset as in Figure 2 (red) and from simulated particle responses with a number of binders that were Poisson distributed (blue). Only particles that had a sufficient number of data points (n ≥ 5) and a good quality fit (adjusted $R^2 \geq 0.8$) were included (N = 881). Time traces of simulated particles were obtained using the following parameters: $k^*_{on} = 5 \cdot 10^{-3}$ s^{-1}, $k_{off} = 0.2$ s^{-1}, $k_{on} = 10^5$ $M^{-1}s^{-1}$, λ_{PSB} = 3 and λ_{SSB} = 5.

S6. Classification of individual particle responses by Fourier analysis

We classified the DRC shapes of particles by analyzing the phase shifts per particle. The analysis workflow is illustrated in Figure S6. The discrete Fourier transform of the response data for each particle was computed using the fast Fourier transform algorithm (MATLAB fft function), as shown for a single particle in Figure S6A. The phase of each particle was obtained from the fundamental frequency component (around 0.5 h^{-1}, see Figure S6B). Figure S6C shows the computed inverse Fourier transform (MATLAB ifft function) of the frequency domain data after removing all frequency components except the zero-frequency and the fundamental frequency components, confirming the selection of the correct frequency component. Phase shifts were obtained by extracting the computed phase from the first DRC of the particle ensemble mean response and subtracting it from the individual particle phases (see Figure S6D).

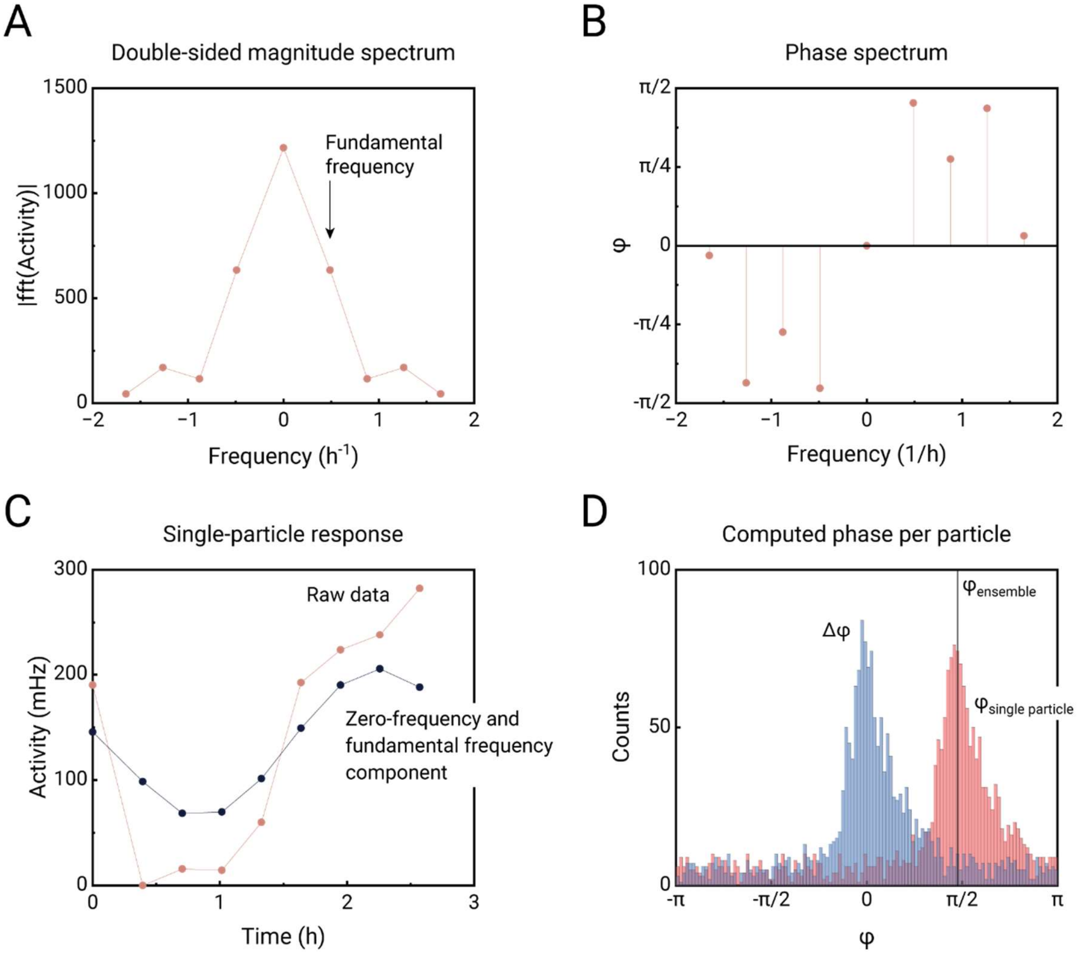

Supporting Figure S6 | Classification of individual particle responses by Fourier analysis. (A) Double-sided magnitude spectrum for the response of a single particle (raw data shown in panel C). (B) Corresponding phase spectrum. (C) Raw response data and the same response data after computing an inverse Fourier transform with the zero-frequency and fundamental frequency component. (D) Computed phases for all particles, showing the computed phases after Fourier transform in red and the computed phase for the first DRC of the particle ensemble mean as the black line. The resulting calculated phase shift is shown in blue, which is essentially the red distribution shifted to the left. The slight differences in shape of the histograms are caused by the redistribution of values into different bins.

S7. Examples of individual particle responses in different phase-shift bins

Figure 4C shows the averaged DRC behavior of particles in different phase-shift bins. In this Section, we show that the indicated average behaviors can also be seen in individual particles. Figure S7 shows the responses of six individual particles. The orange curves were measured on three particles that started with a sigmoidal-shaped DRC. The green curves were measured on three particles that started with a bell-shaped DRC. The graphs show that individual particles exhibit similar changes over time as the ensemble mean of their respective bins: particles with a sigmoidal DRC gradually decrease in amplitude, while particles with a bell-shaped DRC gradually display sigmoidal characteristics. The observed variations on the signals of individual particles can be attributed to temporal heterogeneities, relating to the stochastic nature of the underlying molecular interactions.

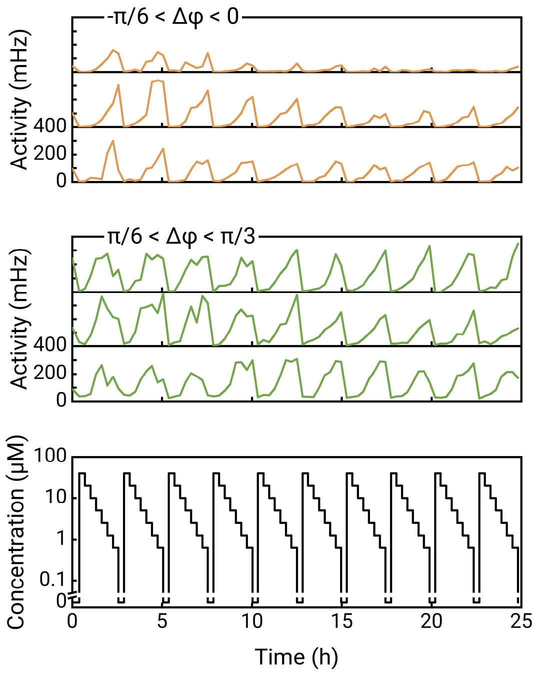

Supporting Figure S7 | Examples of individual particle responses in different phase-shift bins. The orange curves were measured on three particles having phase shifts π/6 < Δφ < 0 in the first DRC (0-2.5h), i.e., a sigmoidal-shaped DRC. The green curves were measured on three particles having phase shifts π/6 < Δφ < π/3 in the first DRC (0-2.5h), i.e., showing a bell-shaped DRC.

S8. Changes of binding valency over long time spans

Figure 4 shows how the responses of single-particle transducers change as a function of time. The changes were attributed to gradual losses of binder molecules from particles and/or substrate. The binder loss hypothesis has been studied in previous work on BPM sensors.[6] In this Section, we corroborate the binder loss hypothesis by analyzing experimentally determined distributions of bound-state lifetimes, and by performing simulations of particle responses with the incorporation of a binder loss rate.

Particles exhibit longer bound-state lifetimes when the binding between particle and substrate is multivalent rather than monovalent. This principle is visible in the simulations of Figure 3B, where the particle bound-state lifetimes increase with increasing numbers of binder molecules, caused by the presence of multivalent binding between particle and substrate. Figure S8A shows measured cumulative distribution functions of bound-state lifetimes of particles with bell-shaped DRCs (phase-shift bin $\pi/6 < \Delta\phi < \pi/3$, in Figures 4B and 4C) in the absence of analyte. The bound-state lifetimes are seen to progressively decrease over time. The decrease is attributed to a gradual transition from multivalent to monovalent binding between particle and substrate, caused by gradual losses of binder molecules on particle and/or substrate.

Figure S8B shows simulation results of particle responses with the incorporation of a gradual loss of binder molecules from the particle. The loss of binders was modelled as a Poisson point process where each individual binder had a lifetime sampled from an exponential distribution with a mean lifetime $1/k_{\text{loss}}$, where k_{loss} represents the average loss rate of binder molecules per particle. Figure S8B shows simulated sensor responses for different values of k_{loss}. For simplicity, the loss rate of SSBs was set to 0, i.e., no SSBs are lost over time. In Figure S8B, the simulation data of particle responses over time show that the changes of response behavior are stronger in case of higher binder loss rates. The results show a transition of bell-shaped responses to sigmoidal responses, as well as a decrease of the amplitude over time, similar to what was observed experimentally in Figures 2 and 4.

Losses of binder molecules affect the analytical performance of the sensor due to effects on signal magnitude and binding valency, for example.[1,7] In experiments we have seen that the observed binder loss rates can vary, depending for example on the used sensor materials (e.g., type of binder molecules, type of particle and substrate), preparation protocols (e.g. coupling methods, buffer compositions) and the sensing conditions (e.g., fluid composition). The underlying mechanisms will be further studied in subsequent research.

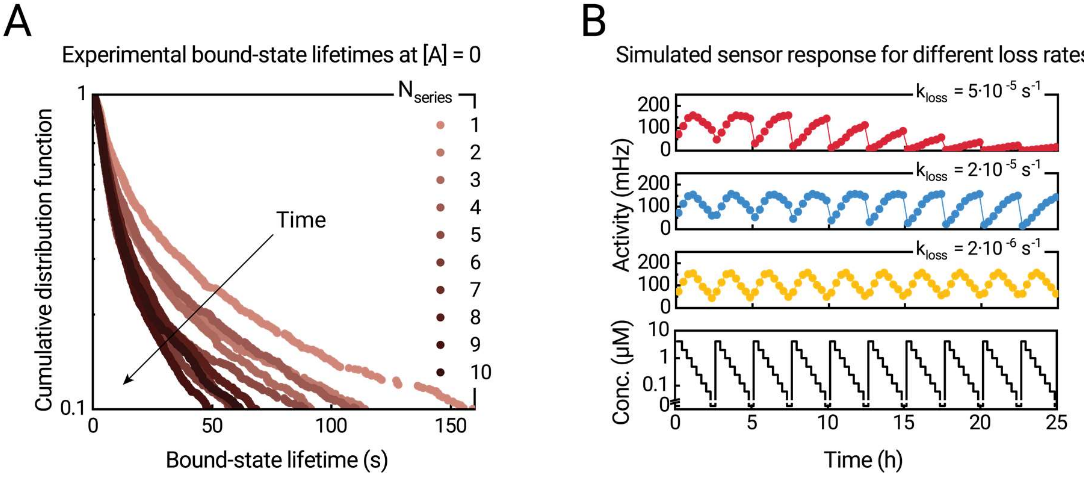

Supporting Figure S8 | Changes of binding valency over long time spans. (A) Experimentally determined bound-state lifetime survival curves in the absence of analyte of particles that exhibited a bell-shaped DRC for the first applied concentration series (same dataset as shown in Figure 4B and 4C, $\pi/6 < \Delta\varphi < \pi/3$). (B) Simulated sensor responses for different PSB loss rates k_{loss}. Results were obtained using the following parameters: $k^{*}_{on} = 5\cdot10^{-3}$ s^{-1}, k_{off} = 0.2 s^{-1}, $k_{on} = 10^{5}$ M^{-1}s^{-1}, N_{PSB} = 20, N_{SSB} = 10. The loss rate for SSBs was set to 0.

S9. Dependency of activity on the number of binder molecules

Figure S9A shows simulated results of how different combinations of N_{PSB} and N_{SSB} lead to different switching activities, calculated without analyte in solution. When increasing the number of binder molecules on one side, the activity first increases, then reaches a maximum, and subsequently decreases toward zero. The decrease occurs when multivalent bonds start to dominate the particle bound states. The number of PSBs needed to reach multivalency depends on the number of SSBs. When the activity is plotted as a function of the binder number product, i.e., the product of N_{PSB} and N_{SSB}, all curves collapse into one single universal curve, see Figure S6B. This indicates that the binder number product $N_{PSB} \cdot N_{SSB}$ is a key parameter for describing the behavior of the particle-based transducer.

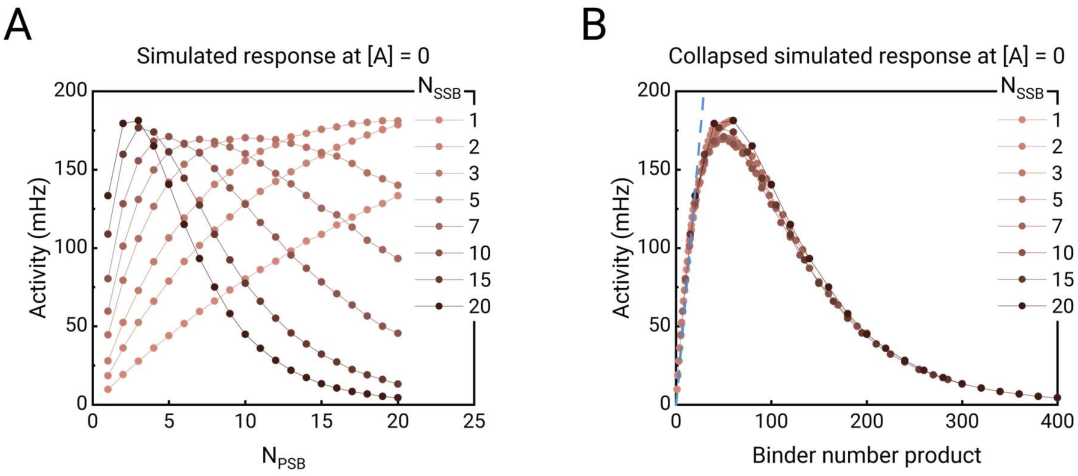

Supporting Figure S9 | Dependency of activity on the number of binder molecules. (A) Simulated switching activities in the absence of analyte for different N_{PSB} and N_{SSB}. Results were obtained using the following parameters: $k^{*}_{on} = 5\cdot10^{-3}$ s^{-1}, $k_{off} = 0.2$ s^{-1}, $k_{on} = 10^{5}$ M^{-1}s^{-1}. (B) Same data as in panel A, now plotted as a function of the binder number product ($N_{PSB} \cdot N_{SSB}$). The blue line indicates the linear regime of the curve where monovalent bonds dominate the particle bound states.

S10. References

1. C. Vu, Y.-T. Lin, S. R. R. Haenen, J. Marschall, A. Hummel, S. F. A. Wouters, J. M. H. Raats, A. M. de Jong, J. Yan, M. W. J. Prins, *Anal. Chem*. **2023**, *95*, 7950–7959.

2. M. H. Bergkamp, L. J. van IJzendoorn, M. W. J. Prins, *ACS Omega* **2021**, *6*, 17726–17733.

3. R. M. Lubken, A. M. de Jong, M. W. J. Prins, *Nano Lett*. **2020**, *20*, 2296–2302.

4. A. D. Buskermolen, Y.-T. Lin, L. van Smeden, R. B. van Haaften, J. Yan, K. Sergelen, A. M. de Jong, M. W. J. Prins, *Nat. Commun*. **2022**, 13, 6052.

5. W. S. Tan, A. M. de Jong, M. W. J. Prins. *ACS Appl. Mater. Interfaces* **2024**, *16*, 58191–58202.

6. S. Cajigas, A. M. de Jong, J. Yan, M. W. J. Prins, *ACS Sens*. **2024**, *9*, 3520–3530.

7. R. M. Lubken, Y.-T. Lin, S. R. R. Haenen, M. H. Bergkamp, J. Yan, P. A. Nommensen, M. W. J. Prins, *ACS Sens*. **2024**, *9*, 4924-4933.
